# Enhanced Infrared Shielding Function in ATO-Doped Polymer-Dispersed Liquid Crystal Films

**DOI:** 10.3390/molecules30081730

**Published:** 2025-04-11

**Authors:** Hongren Chen, Baohua Yuan, Xiao Wang, Xiaoming Zhang, Qilei Wang, Zuowei Zhang, Yunxiao Ren, Yihai Yang, Zihui Ye, Ruochen Lan, Lanying Zhang, Wei Hu, Yong Jiang, Huai Yang

**Affiliations:** 1 School of Materials Science and Engineering, University of Science and Technology Beijing, Beijing 100083, China; hongren8000@163.com (H.C.);; 2 Institute for Advanced Materials and Technology, University of Science and Technology Beijing, Beijing 100083, China; 3 Research Centre for Modern Police Technology and Equipment, China People’s Police University, Langfang 065000, China; 4 School of Materials Science and Engineering, Peking University, Beijing 100871, China; 5 School of Chemical Engineering, Jiangxi Normal University, Nanchang 330022, China

**Keywords:** PDLC, ATO, nanoparticles, infrared shielding

## Abstract

The enhanced infrared shielding function of antimony tin oxide (ATO)-doped polymer-dispersed liquid crystal (PDLC) film enables its use for smart windows, because it can switch between transparent and scattered states, which can protect people’s privacy. When PDLC film is used for a building’s doors and windows or external walls, we hope that it can have a higher infrared shielding capability, in order to reduce the indoor temperature affected by solar irradiation, so as to reduce the energy consumption caused by refrigeration equipment. However, the infrared shielding capability of the existing PDLC is far from sufficient. In this work, modified ATO nanoparticles of different sizes were introduced into the PDLC system to improve its infrared shielding capability. It was found that when the ATO particle size is 20 nm and the doping content is 0.6 wt%, the modified PDLC sample provides optimal infrared shielding function while maintaining excellent electro-optical properties.

## 1. Introduction

Amid growing global concerns regarding environmental sustainability and energy efficiency, the development and implementation of eco-friendly intelligent technologies have emerged as a prominent research frontier [1,2]. Notably, next-generation smart window systems employing electrochromic, thermochromic, gasochromic, and photochromic materials demonstrate exceptional optical modulation capabilities for regulating solar irradiation [3,4,5,6,7,8,9,10,11]. Within this technological landscape, polymer-dispersed liquid crystal (PDLC) systems have gained substantial traction as a mainstream solution due to their exceptional scalability, operational durability, mechanical flexibility, and robust structural integrity [12,13,14,15,16,17].

The fundamental architecture of PDLC composites comprises microdomains of liquid crystal droplets randomly dispersed within a continuous polymer matrix [18,19,20,21]. The operational principle relies on the electric field-induced realignment of LC directors within these microdomains, thereby enabling the dynamic modulation of optical transmittance [22,23,24]. In the voltage-off state, pronounced refractive index mismatch between the anisotropic LC medium and isotropic polymer matrix induces strong light scattering, rendering the film opaque. The application of an external electric field induces uniform LC molecular orientation, achieving refractive index matching and consequent transparent state conversion. This unique electro-optical switching behavior, coupled with the polymer matrix’s inherent chemical stability and mechanical resilience, positions PDLC technology as a promising candidate for advanced smart window applications [25]. In addition, for specific needs, additional PDLC functions such as infrared shielding can be customized, which can achieve more efficient energy-saving effects without affecting the basic functions of PDLC [26,27,28].

One of the most thoroughly investigated systems within the field of PDLCs is the liquid crystal/polymer/nanoparticle system. This tri-component system has garnered significant attention due to its unique properties and potential applications [29,30,31,32,33,34,35]. Current research endeavors predominantly concentrate on enhancing PDLC’s electro-optical parameters, particularly driving voltage minimization, contrast ratio (CR) optimization, and switching time acceleration. Busbee et al. systematically investigated functionalized SiO_2_ nanoparticle (NP) dispersion in holographic PDLC systems, revealing that high-refractive-index nanoparticles amplify the LC/polymer refractive index contrast, thereby enhancing CR values [36]. Furthermore, Yaroshchuk et al. demonstrated that TiO_2_ nanoparticle incorporation not only improves optical contrast but also significantly reduces off-axis haze through optimized light scattering management [34]. Oğuz Köysal et al. investigated CdSeS/ZnS quantum dots doped at varying concentrations in polymer-stabilized liquid crystals (PSLCs) and extracted key physical parameters, including threshold voltage, dielectric anisotropy, relaxation frequency, and response time, from experimental data [37].

In this work, functionalized antimony tin oxide (ATO) NPs of three different sizes (10 nm, 20 nm, and 30 nm) were doped into the PDLC system. The effects of ATO NPs of different sizes and contents on the electro-optical properties, micro-morphology, and infrared shielding function of PDLC were systematically investigated.

## 2. Results and Discussion

### 2.1. Functionalization of ATO NPs

**Figure 1:**
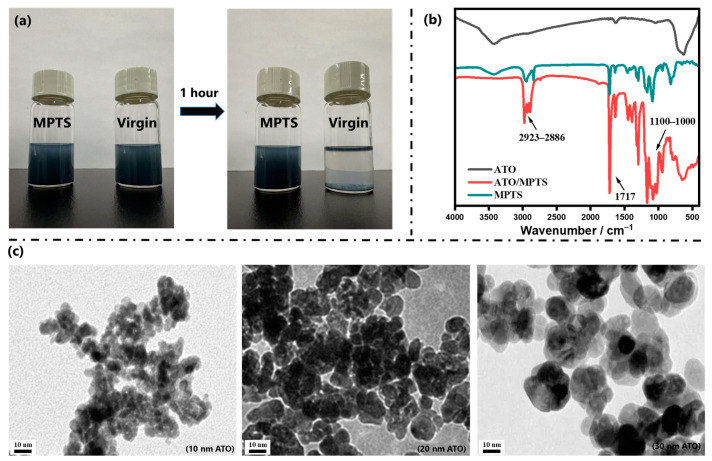
(**a**) Dispersion stability test (virgin vs. MPTS-functionalized ATO NPs in ethanol); (**b**) FT-IR spectra confirming MPTS grafting; (**c**) TEM analysis of ATO NPs of different sizes.

Figure 1a presents comparative digital images of pristine and MPTS-functionalized ATO NP ethanol dispersions under sequential colloidal stability testing. Upon ultrasonic treatment, both systems achieved homogeneous suspensions. Remarkably, after 1 h of quiescent standing, virgin ATO NPs exhibited rapid sedimentation and macroscopic agglomeration, whereas the MPTS-functionalized counterparts maintained stable colloidal dispersion with negligible phase separation. Figure 1b displays comparative FT-IR spectra characterizing the surface modification efficacy. The distinctive spectral signature of MPTS-functionalized ATO NPs reveals three characteristic vibrational modes: (i) prominent absorption bands at 2923–2886 cm^−1^ corresponding to asymmetric stretching vibrations of C-H bonds in methylene (-CH_2_-) and methyl (-CH_3_) groups; (ii) a pronounced peak at 1717 cm^−1^ attributable to C=O stretching vibrations from ester carbonyl groups; and (iii) emergent Si-O-Si stretching modes at 1100–1000 cm^−1^. These spectral features unequivocally confirm the successful covalent grafting of MPTS molecules onto ATO surfaces. Figure 1c demonstrates the morphological evolution through high-resolution TEM micrographs of functionalized ATO nanoparticles with controlled dimensional variations. Notably, the functionalized ATO NPs exhibited substantially improved interparticle spacing and a reduced aggregation tendency. This enhanced dispersibility, coupled with the observed surface roughness in TEM images, provides compelling evidence for the formation of MPTS-derived organosilane layers covalently grafted onto nanoparticle surfaces.

### 2.2. Electro-Optical Properties of PDLC Films

The electro-optical performance of PDLC films is governed by key parameters including threshold voltage (*V_th_*), saturation voltage (*V_sat_*), response time (τ_on_ + τ_off_), and contrast ratio (*CR*). Specifically, *V_th_* and *V_sat_*—defined as the minimum voltages required to attain 10% and 90% of maximum transmittance, respectively—follow inverse proportionality to the liquid crystal’s dielectric anisotropy (Δε) and droplet radius *R*, as expressed by

(1) Vth≈dRKω2−1ε0∆ε12

(2) Vsat≈dRω2−1124πK∆ε

where *d* denotes film thickness, *K* the elastic constant, *ω* the droplet aspect ratio, and *ε₀* vacuum permittivity.

The temporal response is characterized by rise time (*τ*_on_: 10–90% transmittance activation) and decay time (*τ*_off_: 90–10% transmittance relaxation), with a shorter total response time (*τ*_total_) indicating faster switching. Theoretically, they can be calculated according to the following equations:

(3) $$\tau_{\mathrm{on}}\approx \frac{\gamma }{\Delta \varepsilon V^{2}-\frac{K\left(l^{2}-1\right)}{R^{2}}}$$

(4) $$\tau_{\mathrm{off}}\approx \frac{R^{2}\gamma }{K\left(l^{2}-1\right)}$$

where *V* is the applied electric field, while *K*, *l*, *γ*, and Δ*ε* represent the elastic constant, shape anisotropy, rotational viscosity constant, and dielectric anisotropy of the LC, respectively.

The contrast ratio (*CR* = *T_on_*/*T_off_*), determined by the steady-state transmittance ratio between activated (*T_on_*) and relaxed (*T_off_*) states, quantifies optical modulation depth. Optimal PDLC films for practical applications require the coordinated optimization of low driving voltages (*V_th_* < 20 V, *V_sat_* < 50 V), a high *CR* (>20), and subsecond response (τ_total_ < 500 ms), achieved through precise control of LC material properties (Δ*ε*, *K*), droplet morphology (*R*, *ω*), and device architecture (*d*).

**Figure 2:**
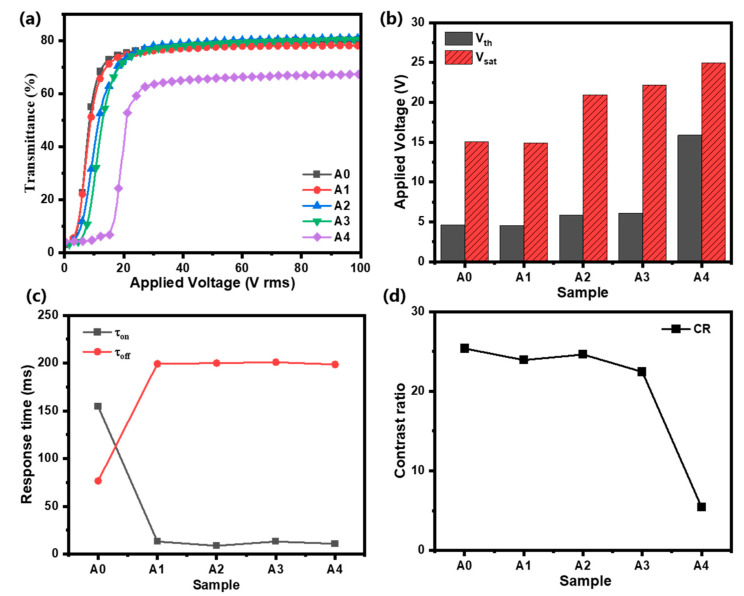
(**a**) The transmittance curve with voltage; (**b**) threshold voltage (*V_th_*) and saturation voltage (*V_sat_*); (**c**) contrast ratio (*CR*); and (**d**) rise time (τ_on_) and decay time (τ_off_) of Samples A0–A4.

The voltage dependence of the transmittance of Samples A0–A4 in Figure 2a shows that with an increase in the applied voltage, the *T_on_* of all samples reaches the saturation level. Figure 2b shows that Samples A0 and A1 have the lowest driving voltages, and as the content of 10 nm ATO NPs increases, the *V_th_* and *V_sat_* of the PDLC films increase. On the one hand, the introduction of NPs changes the size of the polymer mesh, which brings about an effect on the driving voltage, as shown in Equations (1) and (2). On the other hand, the introduction of ATO NPs increases the roughness of the PDLC interface. The larger the particle size of nanoparticles, the greater the influence of doping on roughness [38,39]. The increased anchoring force of the interface to the liquid crystal molecules is induced by the roughness change, which also has an effect on the driving voltage. In summary, the introduction of nanoparticles usually has a negative effect on the driving voltage of PDLC films, which leads to an increase in the driving voltage. Figure 2c shows that compared to Sample A0, Samples A1–A4 have significantly lower τ_on_ values, while τ_off_ increases substantially. According to Equations (3) and (4), since the surface anchoring of the polymer matrix becomes weaker in structures with larger pore sizes, an increase in the liquid chromatography droplet size leads to a decrease in τ_on_ and an increase in τ_off_. In Figure 2d, the CR of Samples A0–A4 gradually decreases, and correspondingly, the T_on_ of Samples A0–A4 also decreases with the introduction of ATO NPs, as shown in Figure 2a. It can be said that the doping of ATO NPs into the PDLC system makes the open-state transmittance of the film decrease.

**Figure 3:**
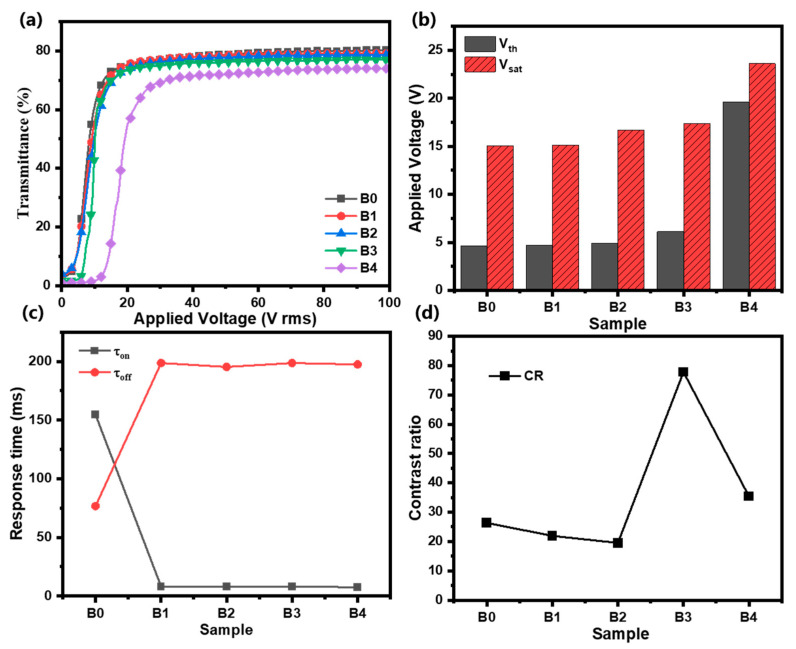
(**a**) The transmittance curve with voltage; (**b**) threshold voltage (*V_th_*) and saturation voltage (*V_sat_*); (**c**) contrast ratio (*CR*); and (**d**) rise time (τ_on_) and decay time (τ_off_) of Samples B0–B4.

**Figure 4:**
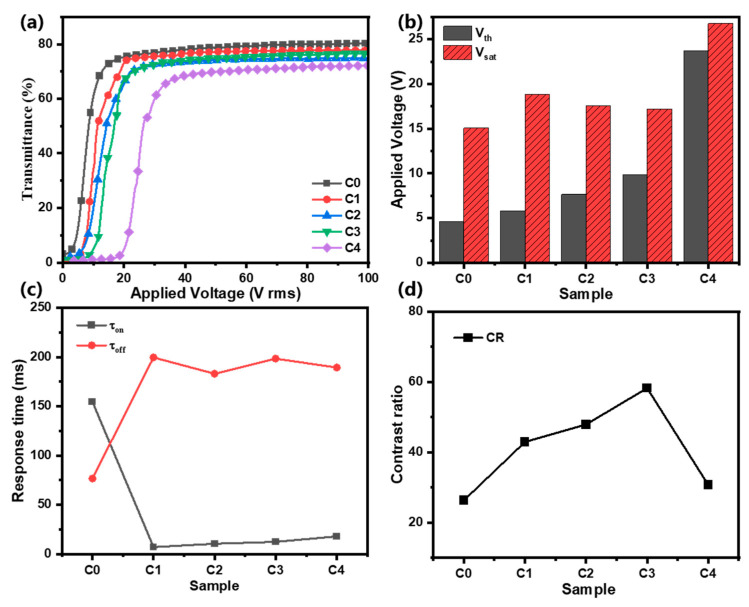
(**a**) The transmittance curve with voltage; (**b**) threshold voltage (*V_th_*) and saturation voltage (*V_sat_*); (**c**) contrast ratio (*CR*); and (**d**) rise time (τ_on_) and decay time (τ_off_) of Samples C0–C4.

In Group B, instead of 10 nm ATO NPs, 20 nm ATO NPs were doped into the PDLC system. In Figure 3a, with an increase in the applied voltage, the T_on_ of Samples B0–B4 reaches the saturation level. As with Group A, the doping of 20 nm ATO NPs into the PDLC system also makes the open-state transmittance of the film decrease. Compared to Samples B0–B2, the off-state transmittance of Samples B3 and B4 is significantly optimized. However, when the concentration of ATO nanoparticles in the film exceeds a threshold level, the open-state transmittance of Sample B4 significantly decreases. In Figure 3b, Samples B0 and B1 have the lowest driving voltages, and as the content of 20 nm ATO NPs increases, the *V_th_* and *V_sat_* of the PDLC films increase. Figure 4c shows that, in contrast to Figure 3c, the increase in ATO NP size does not have an additional effect on the response time of the PDLC films. As shown in Figure 3d, despite the reduction in the open-state transmittance, the reduction in the off-state transmittance leads to a substantial increase in CR for Samples B3 and B4.

In Group C, when the size of ATO NPs continued to be increased to 30 nm, the electro-optical properties of the PDLC films, again, varied. In Figure 4a, with an increase in the applied voltage, the T_on_ of Samples C0–C4 reaches the saturation level. In a comparison with Groups A and B, the open-state transmittance of Group C samples undergoes a significant decrease with the same concentration of ATO NPs. This suggests that an increase in the size of ATO nanoparticles within PDLC films leads to enhanced light scattering efficiency, thereby improving the overall optical performance of the material. In Figure 4b, the driving voltages of Samples C0–C4 show a trend of increasing, then decreasing, and finally increasing again. This is related to the effect of 30 nm ATO NPs on the polymer network morphology of PDLC films, which will be discussed in the next section. In Figure 4c, the off-state response times of Samples C1–C4 show a clear upward trend, which is, again, related to the effect of ATO NP doping on the morphology of PDLC polymer networks. Figure 4d shows that Sample C3 has the highest CR thanks to its low off-state transmittance. Sample C4 also has a low off-state transmittance, but its reduced open-state transmittance results in a low CR.

In summary, the introduction of ATO NPs can effectively reduce the open-state response time of PDLC films and improve the contrast of PDLC films. At the same time, it also negatively affects the driving voltage of the film and improves the off-state response time of the PDLC film. After a comprehensive comparison of the three groups, Sample B3 has the best electro-optical properties. It has a lower driving voltage, *V_th_* and *V_sat_* values of 6.12 V and 17.38 V, respectively, a shorter on-state response time of 8.2 ms, and the highest contrast ratio of 77.8.

### 2.3. Morphology of ATO-Doped PDLC Film

**Figure 5:**
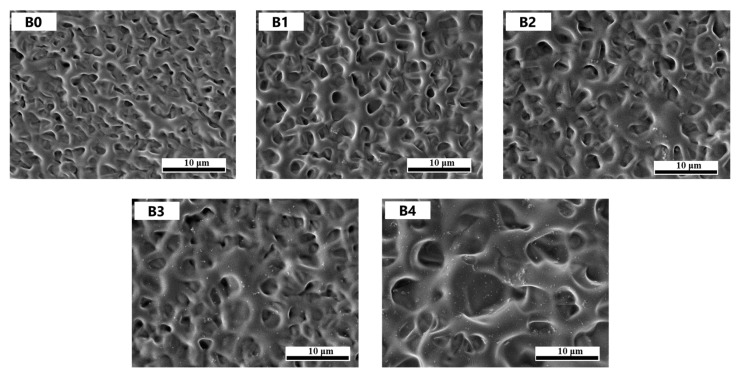
Microphotography of the polymer networks of Samples B0–B4.

As mentioned above, the electro-optical performance of PDLC films is predominantly influenced by two key factors: the morphological characteristics of the polymer matrix and interfacial interactions between liquid crystal (LC) molecules and the polymer network. On this basis, we characterized the microscopic morphology of the Group B samples, as shown in Figure 5. It can be seen that the dimensions of LC droplets undergo an obvious increase in the porous structure with the content of 20 nm ATO NPs increasing from 0.0% to 0.8%. This is because the nanoparticles hinder the propagation of UV light to a certain extent, and the polymerization rate will decrease at a lower UV light intensity, leading to the occurrence of full phase separation to obtain a larger mesh structure. However, only the polymerization rate is affected, and the effect on the polymerization degree is not great when sufficient polymerization time is guaranteed. In addition, it is evident that ATO NPs are uniformly dispersed in the polymer network at a low doping content, but aggregation occurs as the content of ATO NPs continuously increases. Especially in Sample B4, a large number of ATO nanoparticles can be seen clumping together to form larger particles.

### 2.4. Verification of Near-Infrared Shielding Function of ATO-Doped PDLC Film

**Figure 6:**
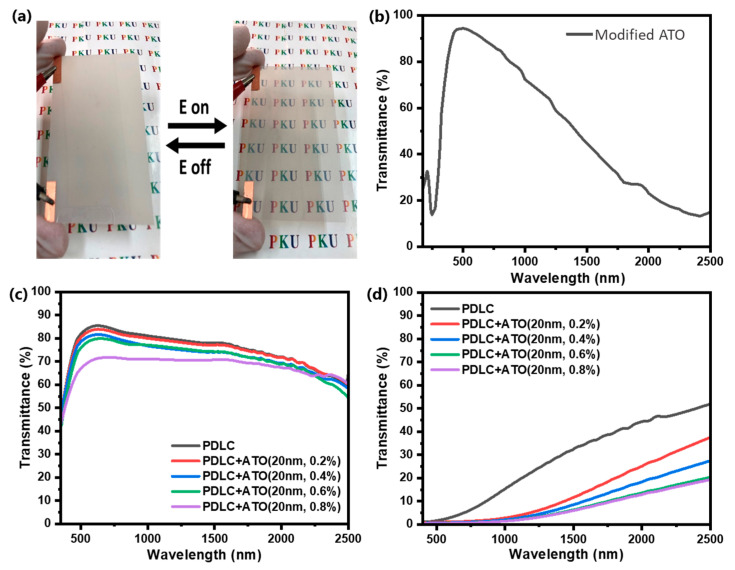
(**a**) PDLC sample sandwiched between two flexible conductive substrates; (**b**) Vis-NIR transmittance spectra of 1 wt% 20 nm modified ATO nanoparticle ethanol solution; (**c**) Vis-NIR transmittance spectra of PDLC samples in on state; (**d**) Vis-NIR transmittance spectra of PDLC samples in off state.

Finally, a 10 cm × 5 cm PDLC film was fabricated to test its near-infrared shielding capability and to demonstrate its potential application in the field of smart displays, as shown in Figure 6. Figure 6a shows that the PDLC sample is sandwiched between two flexible conductive substrates, which can switch between scattering and transparent states under the action of an electric field. The Vis-NIR transmittance spectra of the 1 wt% 20 nm modified ATO nanoparticle ethanol solution from 400 nm to 2500 nm are shown in Figure 6a, which confirms that ATO has very good visible light transmittance and infrared shielding capability. The Vis-NIR transmittance spectra of PDLC film with different doping contents of modified ATO (0%, 0.2%, 0.4%, 0.6%, 0.8%) in the on state and off state of PDLC from 400 nm to 2500 nm are shown in Figure 6c and Figure 6d, respectively. It can be seen that whether in the on state or off state, the introduction of ATO significantly reduces the transmittance of infrared light. When the ATO doping amount exceeds 0.6%, a continuous increase in the doping amount no longer has an obvious infrared shielding effect. However, the visible light transmittance of the sample film is significantly affected, and the transmittance of both on and off states decreases significantly. Therefore, PDLC film with 0.6% modified ATO has optimal overall performance.

## 3. Experimental Section

### 3.1. Materials

**Figure 7:**
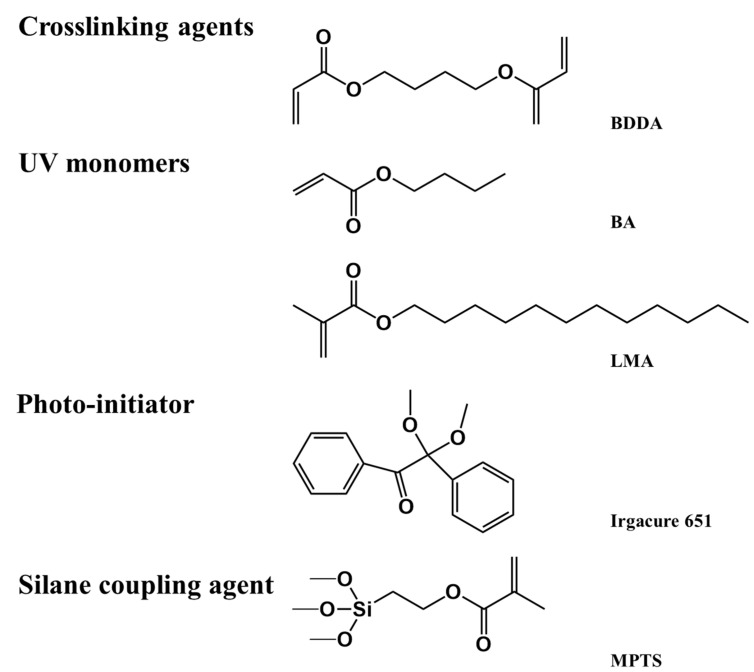
Chemical structure of the materials used in this paper.

The UV monomers 1,4-butanedioldicrylate (BDDA), butyl acrylate (BA), and lauryl methacrylate (LMA) were purchased from Tokyo Chemical Industry Co., Ltd., Shanghai, China. The photo-initiator Irgacure 651 was purchased from Beijing Kaiguo Technology Co., Ltd., China. SLC1717 is a type of commercial nematic liquid crystal that was purchased from Yantai Xian Hua ChemTech. Co., Ltd., Yantai, China. 3-Methacryloxypropyltrimethoxysilane (MPTS) was purchased from J&K Scientific Ltd., Beijing, China. ATO NPs with different particle sizes of 10 nm, 20 nm, and 30 nm were purchased from Zhongke Yannuo Technology Co., Ltd., Beijing, China. The above materials were used without further purification. The chemical structures of UV monomers and the photo-initiator are shown in Figure 7.

### 3.2. Functionalization of ATO NPs

The functionalization of ATO NPs was achieved via silane coupling treatment using MPTS. Specifically, 5 g of MPTS was ultrasonically dispersed (40 kHz, 300 W) in a 500 mL binary solvent system (H_2_O/EtOH, *v*/*v* = 1:1) for 30 min. Subsequently, 1 g of ATO NPs was introduced into the solution under continued ultrasonication (10 min) to ensure colloidal stabilization. The suspension underwent 24 h of mechanical agitation (600 rpm) at 60 °C to promote covalent grafting, followed by vacuum drying (60 °C, 12 h) to yield MPTS-modified ATO NPs. Residual silane impurities were eliminated through three successive washing cycles: (1) ethanol rinsing with 10 min ultrasonication, (2) vacuum filtration, and (3) final vacuum drying at 60 °C.

### 3.3. Preparation of the PDLC Films

Firstly, LC and polymerizable monomers were mixed in the proportions shown in Table 1. Subsequently, the sample was shaken and stirred while heating until the mixed solution showed an isotropic transparent state. Then, the mixture was sandwiched between two transparent conductive polyester substrates via capillary action. Finally, the samples were cured under UV light (365 nm, 10.0 mW/cm^2^) for 10 min. After preparation, PDLC films exhibit a strong light scattering state due to the disordered orientation of the LC molecules dispersed in the polymer matrix.

The fabrication protocol commenced with the precise stoichiometric blending of liquid crystal (LC) and polymerizable monomers according to the compositional ratios specified in Table 1. The homogeneous mixture underwent thermal equilibration at 120 °C under continuous vortex agitation until achieving isotropic phase transparency, as verified by polarized optical microscopy. Subsequent device assembly employed capillary-assisted self-assembly between parallel-aligned indium tin oxide (ITO)-coated polyethylene terephthalate (PET) substrates, maintaining a controlled interlayer spacing of 20 µm via calibrated spacer beads. Photopolymerization was then executed under ultraviolet irradiation (λ = 365 nm; intensity = 10.0 mW cm^−2^) for 10 min, ensuring uniform crosslinking density across the active layer.

### 3.4. Measurements

Morphological analysis: polymer network topology was analyzed via field-emission SEM (Hitachi S-4800, Hitachi, Ltd., Tokyo, Japan) using solvent extraction preparation: (1) 7-day cyclohexane immersion (25 °C) to remove LC constituents; (2) vacuum drying (80 °C, 12 h); and (3) 15 nm Au sputter-coating for charge dissipation.

Electro-optical properties analysis: A liquid crystal parameter analyzer (LCT-5016C, Changchun Liancheng Instrument Co. Ltd., Changchun, China) quantified switching behavior under 560 nm halogen illumination. Transient transmittance (normalized to blank LC cell = 100%) was recorded via a photodiode-coupled oscilloscope under a 100 Hz square-wave AC field. Response kinetics (rise/fall times) were derived from 10 to 90% transmittance transitions.

UV–VIS-NIR spectrum analysis: The spectral properties of the PDLC films were quantified using a PerkinElmer Lambda 950 (PerkinElmer Inc., Waltham, MA, USA) ultraviolet–visible–near-infrared (UV-VIS-NIR) spectrophotometer. Spectral transmittance data were acquired across 400–2500 nm, from which two critical metrics were derived:

(5) $$T_{sol}=\frac{\int_{400}^{2500}T\left(\lambda \right)\;\varphi sol\left(\lambda \right)\;d_{\lambda }}{\int_{400}^{2500}\;\varphi sol\left(\lambda \right)\;d_{\lambda }}$$

where *T(λ)* represents wavelength-dependent transmittance, and *φ* sol(λ) is the solar irradiance spectrum distribution for air mass 1.5 corresponding to the sun standing 37 °C above the horizon with 1.5 atm thickness at a solar zenith angle of 48.2° [40].

## 4. Conclusions

In conclusion, we prepared a PDLC film with enhanced infrared shielding function by introducing modified ATO nanoparticles of different sizes into the PDLC system to improve its infrared shielding capability. It was found that ATO nanoparticles have good infrared shielding ability, but when the ATO particle size is too large, it will significantly affect the transmittance of the visible light band. In addition, when the ATO doping amount is too large, the nanoparticles will agglomerate significantly, which will have a negative effect on performance. Therefore, when the ATO particle size is 20 nm and the doping content is 0.6 wt%, the modified PDLC sample provides optimal infrared shielding function while maintaining excellent electro-optical properties.

## Figures and Tables

**Table 1:** The compositions (wt.%) of all samples.

Samples	Monomers	ATO (10 nm)	ATO (20 nm)	ATO (30 nm)	SLC1717
	BDDA/BA/LMA (1/2/2)				
Group1:					
A0	30.0	0.0			70.0
A1	30.0	0.2			69.8
A2	30.0	0.4			69.6
A3	30.0	0.6			69.4
A4	30.0	0.8			69.2
Group2:					
B0	30.0		0.0		70.0
B1	30.0		0.2		69.8
B2	30.0		0.4		69.6
B3	30.0		0.6		69.4
B4	30.0		0.8		69.2
Group2:					
C0	30.0			0.0	70.0
C1	30.0			0.2	69.8
C2	30.0			0.4	69.6
C3	30.0			0.6	69.4
C4	30.0			0.8	69.2

The weight of photo-initiator Irgacure 651 is 0.5% of the monomers.

## Data Availability

The original contributions presented in this study are included in the article. Further inquiries can be directed to the corresponding authors.
